# Supplementary material for: Choreographing rhizosphere effect with agricultural practices for agroecology?
Source: PLoS One. 2025 Sep 4;20(9):e0327301. doi: 10.1371/journal.pone.0327301 (PMC12410807; doi:10.1371/journal.pone.0327301)
Supplement: Attachment — Submitted filename: Response to Reviewers_def.docx [file pone.0327301.s001.docx]

**Authors' reply**

In general, the comments are very short: only one word, and 3 lines of comments, which to our opinion reflect a hastily review. The comments are neither constructive or informative for authors. The anonymity of the reviewing process, that we respect, should not divert from a rigorous review.

We have provided a point-by-point answers to reviewer #1 and Editor’s comments

**Editor's Responses to Questions**

Thank you for submitting your manuscript to PLOS ONE. After careful consideration, we have decided that your manuscript does not meet our criteria for publication and must therefore be rejected.

Specifically: The objectives are ambiguous and the methods used were simply inadequate to answer the research question. The analysis of the data is defective. The conclusions cannot be justified based on the defective experimentation. There are excessive assumptions without any precise experimental findings.  I am sorry that we cannot be more positive on this occasion, but hope that you appreciate the reasons for this decision.

Kind regards,

Peddisetty Pardha-Saradhi, Ph.D.

Academic Editor

PLOS ONE

Additional Editor Comments:

- In general, the manuscript is incomprehensible.
  - Editor wrote that “In general, the manuscript is incomprehensible“. Reviewer had answered to question 4. “Is the manuscript presented in an intelligible fashion and written in standard English?” with a positive answer making unintelligible the editor’s comment about ambiguity or poor quality of redaction. Moreover, this article was internally reviewed by senior collaborators not involved by the experiment to ensure a minimal quality before submission. We reject firmly the term “incomprehensible”. We addressed a point-by-point answers to other comments.
- The objectives are ambiguous and the methods used were simply inadequate to answer the research question.
  - We are very surprise by the use of the term “ambiguous” and “inadequate” methods. We would be happy to discuss with academic editor to know exactly what type of methods he/she has in mind to study rhizosphere effect on farm because according to world-wide expert such methods do not exist. Please refer to reviewer’s comment. We kindly remind to academic editor that we have studied the soil volume influence by living roots not bulk soils. For sake of clarity, the introduction section was modified (see revised version)
- The analysis of the data is defective.
  - In our manuscript, the statistical analysis of the data is based on a linear mixed model, which is a very classical statistical tool in life science and particularly in ecology. In our study, the use of a linear mixed model is particularly appropriate for 2 main reasons. Firstly, we are studying the additivity of individual effects and interaction effets between experimental factors (soil type, level of diversification and type of tillage). Secondly, it is quite natural and very common to consider sampling sites as a random factor, since it is impossible to sample exhaustively on all possible farms : a very large literature is dedicated to the introduction of mixed models since the 90’s.
  - Secondly, we took advantage of the theoretical framework of linear mixed models to analyze in detail the significant effects of the models. This classic framework of post-hoc analysis is also widely used in statistical modeling, and has enabled us to define the various rhizosphere effect indicators (REIs) presented in our manuscript. Thanks to the classical and natural framework of linear mixed models, these indicators have very simple formulas based on the difference between two model parameters. Furthermore, the framework of linear mixed models allows a natural estimation of the REIs as well as their uncertainty notably by calculating their variances.
  - It's important to note that our proposed modeling methodology using linear mixed models and post-hoc analysis has been in use for several decades. As proof, the R packages used in our study (lme4 and emmeans) have become gold standards with a very high number of uses and citations.
  - **We therefore consider that our analysis of data is classical, robust enough to be reproduced by readers and particularly adapted to answer our research question.**
  - **In conclusion, we reject firmly this allegation, and encourage academic editor to suggest alternative statistical methodology.**
- The conclusions cannot be justified based on the defective experimentation.
  - The experimentation is not defective. It was prepared and designed with respect to the hypotheses we wanted to test. The experimentation has also been designed (in terms of sample size and balance between the variability factors) in accordance with the statistical framework expected to be used once data has been acquired.
  - All experimental methods are justified in the introduction section, and repeated in discussion for sake of integrity. We had particularly justified our choices dealing with the analysis of microbial diversity from a companion paper (Mira et al, 2022), please refer to reviewer’s answer.
  - **We therefore consider that the conclusions are drawn upon a very robust statistical analysis of well-designed experimentation.**
- There are excessive assumptions without any precise experimental findings.
- We would be happy to discuss on which exactly assumptions are estimated excessive.
- Avoid making the manuscript fearsome through apparently complex formulae.
  - The formalism used in our manuscript is limited to the expression of a linear mixed model... The formulae is not complex, on the contrary it is a very classical formulae. It simply makes explicit all the effects we have tested in our method, and thus introduces the parameters that will be used to define the statistical indicators of the rhizoshphere effect (REI).
  - Secondly, we formally define the REI (Rhizosphere Effect Indicators) proposed in our study. These indicators are just a difference between two coefficients of the linear mixed model, and we consider that a difference between 2 terms is not a complex formula. On the contrary, we believe that these formulae allow a better understanding of the construction of our indicators and facilitate the interpretation of the tables and figures presented. Our formulae give a very clear use of our mixed linear modeling and provide evidence for the appropriate statistical analysis conducted in our study.
  - Moreover, we consider the comment “Avoid making the manuscript fearsome through apparently complex formulae” as unacceptable, because mathematical complexity should never be a criterion for rejecting an article.
  - The research gate profile of academic editor is not perfectly aligned with objectives of the submitted article, which appeal to more caution about decision if based solely on reviewer’s comment which we, authors, considered not rigorous.

**Reviewer's comments**

**Comments to the Author**

1. Is the manuscript technically sound, and do the data support the conclusions?

The manuscript must describe a technically sound piece of scientific research with data that supports the conclusions. Experiments must have been conducted rigorously, with appropriate controls, replication, and sample sizes. The conclusions must be drawn appropriately based on the data presented.

Reviewer #1: No

- We would like to know why exactly reviewer#1 answered “no” without pointing out some precise elements from our manuscript to support her/his decision. Using the editorial policies of PlosOne we addressed a point-by-point answer to reviewer#1.
- Rigorously.
  - The analysis was performed according all international procedures.
  - Samples and soil incubations respected all recommendations from literature in terms of transport, storage, pre incubation duration (Thuries et al 2000 cited as reference).
  - Rhizosphere soils were collected according regular methodology commonly accepted by rhizosphere experts. There is no standard procedure for collecting rhizosphere soils.
  - A baseline of enzyme activities of bulk soil was determined to estimate the rhizosphere effect as it influences rhizosphere enzymes, so there are no need to discuss the effect of soil management on soil bulk enzymes as these effects are integrated in the evaluation of the rhizosphere effect.
  - Enzyme assays are considered proxies of microbial activities and are repeatable, reproducible and sensitive to environmental changes or perturbations (Cheviron et al. 2021). Moreover, enzyme assays in micro-well plates can be developed for fast-throughput routine soil analysis.

Cheviron N, Grondin V, Marrauld C, Poiroux F, Bertrand I, Abadie J, Pandard P, Riah-Anglet W, Dubois C, Malý S, Marques CR, Asenjo IV, Alonso A, Díaz DM, Mougin C (2021) Inter-laboratory validation of an ISO test method for measuring enzyme activities in soil samples using colorimetric substrates. Environmental Science and Pollution Research. doi: 10.1007/s11356-021-17173-3.

- Appropriate controls
  - We kindly remind that our experiment is the evaluation of the rhizosphere effect on farms, and the statistical method is adapted accordingly compared to a control experiment where controls are possibles
- Replication.
  - Four replicates per site were determined
  - For enzyme assays, we used 4 technical replicates in addition to the four replicates per site
- Sample size
  - Please refer to elements cited before.

2. Has the statistical analysis been performed appropriately and rigorously?

Reviewer #1: No

- **This comment is unacceptable.**
- In our manuscript, the statistical analysis of the data is based on a linear mixed model, which is a very classical statistical tool in life science and particularly in ecology. In our study, the use of a linear mixed model is particularly appropriate for 2 main reasons. Firstly, we are studying the additivity of individual effects and interaction effects between experimental factors (soil type, level of diversification and type of tillage). Secondly, it is quite natural and very common to consider sampling sites as a random factor, since it is impossible to sample exhaustively on all possible farms : a very large literature is dedicated to the introduction of mixed models since the 90’s.
- Secondly, we took advantage of the theoretical framework of linear mixed models to analyze in detail the significant effects of the models. This classic framework of post-hoc analysis is also widely used in statistical modeling, and has enabled us to define the various rhizosphere effect indicators (REIs) presented in our manuscript. Thanks to the classical and natural framework of linear mixed models, these indicators have very simple formulas based on the difference between two model parameters. Furthermore, the framework of linear mixed models allows a natural estimation of the REIs as well as their uncertainty notably by calculating their variances. Yet statistical novelty of our approach is to fully exploit the statistical properties of the linear mixed models to evaluate a rhizosphere effect taking into account the bulk soils. It is then important to reveal with the different equations the relationships between the two soil compartments.
- It's important to note that our proposed modeling methodology using linear mixed models and post-hoc analysis has been in use for several decades. As proof, the R packages used in our study (lme4 and emmeans) have become gold standards with a very high number of uses and citations.
- **We therefore consider that our analysis of data is classical, robust enough to be reproduced by readers and particularly adapted to answer to our research question. The novelty of our approach is not the linear mixed model by itself, but the exploitation of the statistical properties of these models to build new indicator of the rhizosphere activities.**
- **In conclusion, we reject firmly this allegation, and encourage reviewer#1 to suggest alternative statistical methodology or methodology that also cope with the need of farmers to obtain reliable, low cost measurements of the rhizosphere, stressed by worldwide experts as a vital interface in future for plant productivity.**

3. Have the authors made all data underlying the findings in their manuscript fully available?

The [PLOS Data policy](http://www.plosone.org/static/policies.action#sharing) requires authors to make all data underlying the findings described in their manuscript fully available without restriction, with rare exception (please refer to the Data Availability Statement in the manuscript PDF file). The data should be provided as part of the manuscript or its supporting information, or deposited to a public repository. For example, in addition to summary statistics, the data points behind means, medians and variance measures should be available. If there are restrictions on publicly sharing data—e.g. participant privacy or use of data from a third party—those must be specified.

Reviewer #1: No

- During submission process, we have specified that all data will be available.
- **This answer is accordingly not receivable and could not be use to reject our article.**

4. Is the manuscript presented in an intelligible fashion and written in standard English?

PLOS ONE does not copyedit accepted manuscripts, so the language in submitted articles must be clear, correct, and unambiguous. Any typographical or grammatical errors should be corrected at revision, so please note any specific errors here.

Reviewer #1: Yes

- We thank reviewer #1 for this positive answer, pointing out a need of more in-depth comments from reviewer #1.

5. Review Comments to the Author

Please use the space provided to explain your answers to the questions above. You may also include additional comments for the author, including concerns about dual publication, research ethics, or publication ethics. (Please upload your review as an attachment if it exceeds 20,000 characters)

Reviewer #1: This study attempted to address the effect of tillage practices on enzyme activities in the wheat field. However, there is a lot of literature available in this field regarding changes in microbial diversity. They are only talking about diversification. What about soil properties, microbial diversity, and so on? Also, there is no clear objective of this study. There is a significant amount of speculation in the discussion section. The conclusion section needs to be rewritten to be more precise. The quality of the figures is not good. The study area map must be included.

We address point by point our answers to these comments

1. *“This study attempted to address the effect of tillage practices on enzyme activities in the wheat field.”*

- We think that this commentary is not appropriate. We would like to insist that our title is **“Choreographing rhizosphere effect with agricultural practices for agroecology”** and not “effect of tillage practices on enzymes activites in a wheat field”.

2. *“However, there is a lot of literature available in this field regarding changes in microbial diversity.”*

- We do not understand the logical link with the previous comment. We consider rhizosphere enzymes as others authors as footprint of plant-microbe interactions that can offer two types of information on bulk soil but also on the small soil volume influenced by living roots, the rhizosphere. We kindly remind to reviewer #1, that rhizosphere was defined by Hiltner in 1904, is currently lacking of affordable indicator to tackle the importance of rhizosphere functioning for agroecology due to its inherent complexity (Vetterlein et al 2022).
- To clarify this point we made substantial changes in the manuscript **LL 79-88** in introduction section
- We kindly recommend to reviewer #1 to re-read the first paragraph of the discussion section **(LL 299-303).**

Vetterlein D, Carminati A, Schnepf A (2022) Special issue: Rhizosphere spatiotemporal organisation: an integrated approach linking above and belowground. Plant and Soil 478 (1):1-4. doi:10.1007/s11104-022-05716-7

- Moreover, it seems that reviewer #1 has missed that we are dealing with diversification as a practice in addition to tillage (L80, L99). We have calculated a Crop Diversity Index (table 1) as many articles (Costa et al, 2024; Smith et al 2023, Tiemann et al. 2015) and in particular in Mira et al (2022), a companion paper published by same authors in 2022 (Plant Soil). We have better explained the Crop Diversity Index by renaming it in “Crop Rotation Diversity” index in material and methods **(LL 100)** section, and following sections.
- Finally, we are addressing in this article the combined effect of these practices which can alter the rhizosphere effect. The discussion is tailored accordingly to examine their impacts on rhizosphere effect captured by difference of activities between bulk and rhizosphere enzymes. We would be happy to discuss with reviewer#1 about alternative methodology to capture rhizosphere effect that we previously debated in our published article (Mira et al, 2022) cited by Vetterlein et al, 2022 world-wide experts on rhizosphere ecology in their special issue.

Smith ME, Vico G, Costa A, Bowles T, Gaudin ACM, Hallin S, Watson CA, Alarcòn R, Berti A, Blecharczyk A, Calderon FJ, Culman S, Deen W, Drury CF, Garcia AGy, García-Díaz A, Plaza EH, Jonczyk K, Jäck O, Lehman RM, Montemurro F, Morari F, Onofri A, Osborne SL, Pasamón JLT, Sandström B, Santín-Montanyá I, Sawinska Z, Schmer MR, Stalenga J, Strock J, Tei F, Topp CFE, Ventrella D, Walker RL, Bommarco R (2023) Increasing crop rotational diversity can enhance cereal yields. Communications Earth & Environment 4 (1):89. doi:10.1038/s43247-023-00746-0

Costa A, Bommarco R, Smith ME, Bowles T, Gaudin ACM, Watson CA, Alarcón R, Berti A, Blecharczyk A, Calderon FJ, Culman S, Deen W, Drury CF, Garcia y Garcia A, García-Díaz A, Hernández Plaza E, Jonczyk K, Jäck O, Navarrete Martínez L, Montemurro F, Morari F, Onofri A, Osborne SL, Tenorio Pasamón JL, Sandström B, Santín-Montanyá I, Sawinska Z, Schmer MR, Stalenga J, Strock J, Tei F, Topp CFE, Ventrella D, Walker RL, Vico G (2024) Crop rotational diversity can mitigate climate-induced grain yield losses. Global Change Biology 30 (5):e17298. doi:https://doi.org/10.1111/gcb.17298

Mira S, Mougel C, Ourry M, Le Cadre E. A field indicator for rhizosphere effect monitoring in arable soils. Plant and Soil. 2022.

Tiemann LK, Grandy AS, Atkinson EE, Marin-Spiotta E, McDaniel MD (2015) Crop rotational diversity enhances belowground communities and functions in an agroecosystem. Ecology Letters 18 (8):761-771. doi:https://doi.org/10.1111/ele.12453

3. “They are only talking about diversification. What about soil properties, microbial diversity, …”

- We have provided the soil analysis (table 1). The similar soil properties do not support to consider them as another factor (refer to clay, pH and organic matter content).
- Moreover, to account for the variability due to various sampling locations, the study site was taken as a random factor.
- We have already considered microbial diversity to calculate the rhizosphere effect in Mira et al (2022). However, these data do not improve the accuracy of the rhizosphere indicator. In addition, in our pioneer article (Mira et al 2022), we clearly stated that developing a more functional approach to the Rhizosphere Effect Indicator may also help design crop rotations, that can magnify the rhizosphere effect. However, the rhizosphere indicator must be affordable for farmers, which would not be if microbial analysis were included. To better consider this point, we made substantial changes in the manuscript in introduction (LL 82-88)
- We kindly recommend to re-read paragraph 4.1 of the discussion section with extensively address all the limits for reader’s full understanding (LL 294-316).

4. “… and so on?”

- We apologize to reviewer#1 and academic editor but we are not able to answer to this comment which is not enough detailing the reviewer’s point.

5. Also, there is no clear objective of this study.

- We consider that we can improve the last part of the introduction, so we made changes to better highlight the objectives of the study. The changes are in red in the revised version (LL 82-88) and pasted here below :

*“According to literature, the objective of our study was to determine if management of the rhizosphere is possible through agricultural practices. To address this objective, we hypothesized that (i) crop rotation and (ii) reduced tillage by preserving soil habitat for microbes would enhance the rhizosphere effect. To tackle our objective, we determine a rhizosphere effect based on difference of functioning between bulk and rhizophere. In line with our pioneer article (Mira et al 2022), this indicator is based solely on rhizosphere and bulk soils enzymes to be transferable to farmers and advisors at a moderate cost. We finally collected the farmer’s perception by focus group and analyzed their willing to modify their practice based on rhizosphere effect determination”*

6. There is a significant amount of speculation in the discussion section.

- Without any example issued from our first version, it is difficult to answer to this comment. However, we consider that we can improve discussion, so we carefully reviewed our discussion and all changes are in red in the revised version (LL 344-351). We therefore considered the reviewer#1 perception and we hope that we better stick to our results.

7. The conclusion section needs to be rewritten to be more precise.

- However, we consider that we can improve conclusion, so we made substantial changes to address the perception of the reviewer. The changes are in red (LL 355-358) in the revised version, and pasted hereafter.

*“We demonstrated that the rhizosphere effect can be significantly altered by agricultural practices either soil or cropping system design. Our results point out to revise not only plant breeding programs but also system approach to benefit rhizosphere effect for plant nutrition in a growing concern of non renewable resources for farmers.”*

8. The quality of the figures is not good.

We do not understand this comment without any example issues from our last version. The figures 1 and 2 contains all statistical informations needed, are correctly legended. The format of figures files are png, and once printed quality is good (3721 × 2093 pixels).

9. “The study area map must be included”
   - Our experiment was performed on farms, not on experimental research facilities. Accordingly, we have to respect anonymity of farmers involved in the study, as a participative research project. All data will be available on request to authors as specified during the submission process. In particular, we have detailed the rotation, fertilization practices. For ethical considerations, we cannot reveal the exact geographical coordinates of fields. Finally, we consider that providing a map does not provide additional information to better understand the results and conclusion of the article.

6. PLOS authors have the option to publish the peer review history of their article ([what does this mean?](https://journals.plos.org/plosone/s/editorial-and-peer-review-process#loc-peer-review-history)). If published, this will include your full peer review and any attached files.


If you choose “no”, your identity will remain anonymous but your review may still be made public.


**Do you want your identity to be public for this peer review?** For information about this choice, including consent withdrawal, please see our [Privacy Policy](https://www.plos.org/privacy-policy).

Reviewer #1: No
